# m6A regulator-mediated methylation modification patterns and immune microenvironment infiltration characterization in osteoarthritis

**DOI:** 10.1186/s12920-022-01429-z

**Published:** 2022-12-30

**Authors:** Shidong Hu, Chen Shen, Xudong Yao, Yulong Zou, Ting Wang, Xianding Sun, Mao Nie

**Affiliations:** Center for Joint Surgery, Department of Orthopedic Surgery, The Second Affiliated Hospital of Chongqing Medical University, No. 76 Linjiang Road, Yuzhong District, Chongqing, 400016 China

**Keywords:** RNA N6-methyladenosine, Osteoarthritis, Bioinformatic analysis, Subtype classification, Immune infiltration

## Abstract

Osteoarthritis (OA) is a common disease in orthopedics. RNA N6-methyladenosine (m6A) exerts an essential effect in a variety of biological processes in the eukaryotes. In this study, we determined the effect of m6A regulators in the OA along with performing the subtype classification. Differential analysis of OA and normal samples in the database of Gene Expression Omnibus identified 9 significantly differentially expressed m6A regulators. These regulators were monitored by a random forest algorithm so as to evaluate the risk of developing OA disease. On the basis of these 9 moderators, a nomogram was established. The results of decision curve analysis suggested that the patients could benefit from a nomogram model. The OA sample was classified as 2 m6A models through a consensus clustering algorithm in accordance with these 9 regulators. These 2 m6A patterns were then assessed with principal component analysis. We also determined the m6A scores for the 2 m6A patterns and their correlation with immune infiltration. The results indicated that type A had a higher m6A score than type B. Thus, we suggest that the m6A pattern may provide a new approach for diagnose and provide novel ideas for molecular targeted therapy of OA.

**Supplementary Information:**

The online version contains supplementary material available at 10.1186/s12920-022-01429-z.

## Introduction

Osteoarthritis (OA) is the most prevalent joint disease around the world [1], and it results in enormous socioeconomic medical costs every year [2]. The current OA treatment can only relieve symptoms and X-ray characteristics and lacks an effective drug treatment. Moreover, the treatment outcomes for advanced osteoarthritis are not satisfactory. Therefore, the early diagnosis of arthritis, though difficult at present, is of great significance to patients. The diagnosis of osteoarthritis mainly relies on medical history, symptoms, signs, and imaging methods, but there is a lack of corresponding biomarkers for diagnosis. Therefore, we believe that establishing biomarkers for the diagnosis of osteoarthritis can facilitate the improvement of patient prognosis and provide novel targets for treating OA.

RNA N6-methyladenosine (m6A) is the most prevalent internal modification in the eukaryotic mRNAs- methylated at the N6 site of adenosine [3]. m6A participates in the pathogenesis of a variety of diseases and also mediates RNA generation and its metabolism [4]. The functionality of m6A is primarily through the coregulation of m6A-binding proteins, demethylases, and m6A methyltransferases [5]. Among these, m6A methyltransferases are called “writers”-responsible for installation, demethylases are called “erasers”- responsible for removal, and m6A-binding proteins called “readers”- are responsible for recognition [6].

Recently, various research groups have demonstrated that m6A plays some role in the OA progression [7, 8], along with the immune factors [9]. In this study, we investigated the effect of m6A regulators in the diagnostic classification of osteoarthritis disease and assessed the extent of osteoarthritis immune infiltration. As a result, 9 key regulators were screened from the database to evaluate the disease risk in osteoarthritis while assessing the immune microenvironment of the two m6A patterns of osteoarthritis. The results obtained are suggested to be helpful for the subsequent diagnosis and targeted therapy of osteoarthritis.

## Results

### Expression of 21 m6A regulators in osteoarthritis

**Fig. 1:**
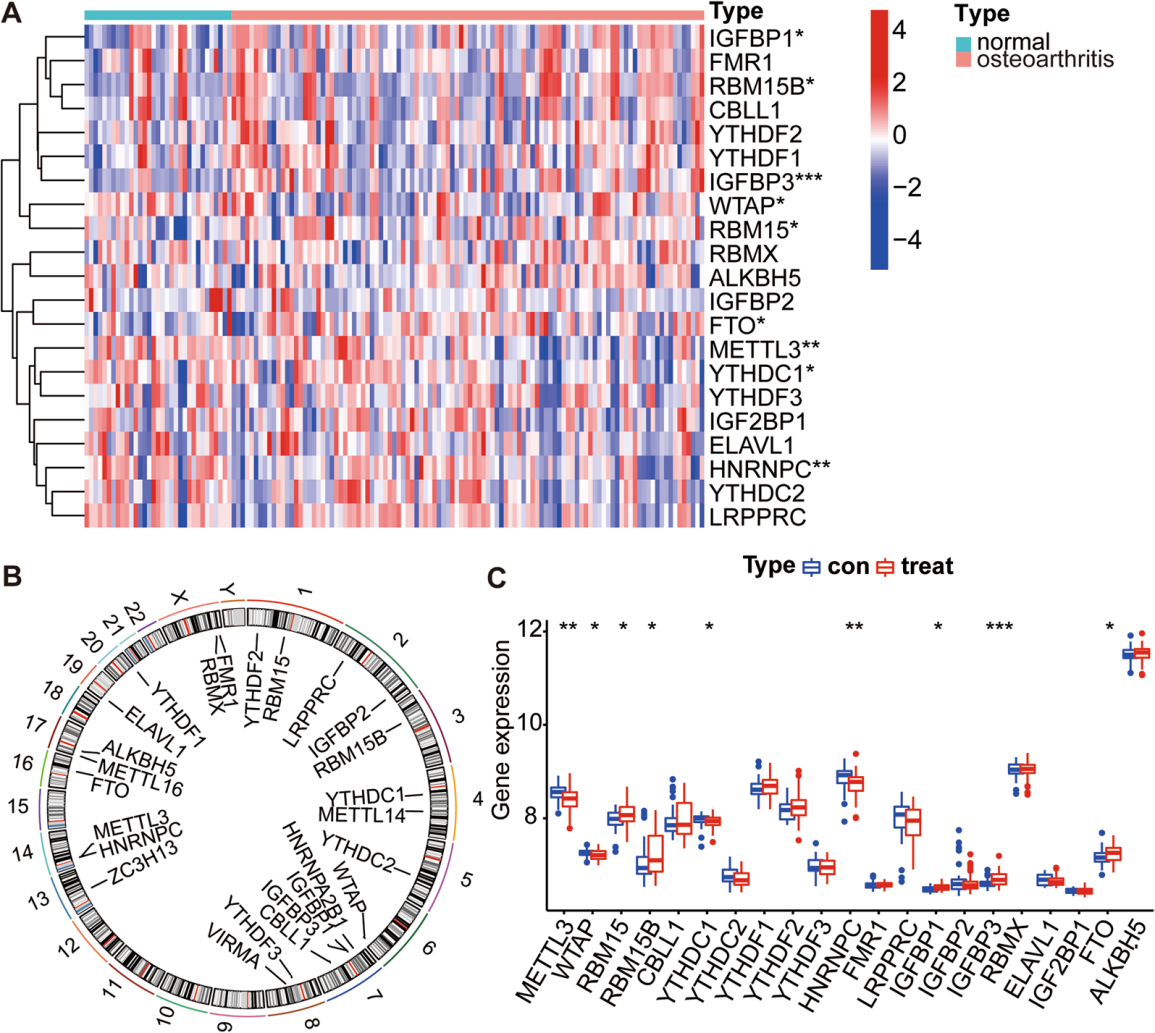
A total of 21 m6A regulators expression in OA. **A** The heatmap of 21 m6A regulators expression between normal and OA samples. **B** Circumference diagram for chromosomal localization in 21 m6A regulators. **C** The histogram of 21 m6A regulators expression between normal and OA samples. *p < 0.05, **p < 0.01, and ***p < 0.001

R software is used to normalize the differential analysis of OA and normal control sample data. The expression values for twenty-one m6A regulators in normal and OA sample data were obtained, and 9 significant differentially expressed regulators (i.e., METTL3, WTAP, RBM15, RBM15B, YTHDC1, HNRNPC, IGFBP1, IGFBP3, and FTO) were screened. All 9 m6A regulators were visualized with heatmaps and histograms, and the differential expression of the IFGBP3 regulator was observed to be relatively significant (Fig. 1A, C). The adjust *p* values and 95% confidence intervals of the differential analysis of the expression levels of the 21 m6A regulators in osteoarthritis and normal specimens are shown in Table 1. Chromosomal localization of each of the 21 m6A regulators was visualized by circos plot (Fig. 1B).

**Table 1:** The *p* value and confidence interval of the differential analysis of the expression of 21 m6A regulators in osteoarthritis and normal specimens

Gene	*P*(adj)	95% CI
METTL3	0.00119585	0.057 to 0.225
WTAP	0.027433004	0.007 to 0.087
RBM15	0.036048033	− 0.184 to -0.005
RBM15B	0.043419108	− 0.325 to − 0.004
CBLL1	0.67212293	− 0.131 to 0.155
YTHDC1	0.040185136	0.004 to 0.096
YTHDC2	0.090938116	− 0.014 to 0.139
YTHDF1	0.139509762	− 0.157 to 0.022
YTHDF2	0.146946623	− 0.163 to 0.026
YTHDF3	0.622338255	− 0.066 to 0.109
HNRNPC	0.002370492	0.055 to 0.213
FMR1	0.383631831	− 0.038 to 0.015
LRPPRC	0.186270112	− 0.039 to 0.231
IGFBP1	0.011998395	− 0.063 to − 0.008
IGFBP2	0.40144281	− 0.033 to 0.087
IGFBP3	0.000871699	− 0.129 to − 0.034
RBMX	0.764569806	− 0.079 to 0.058
ELAVL1	0.536069356	− 0.028 to 0.067
IGF2BP1	0.376912508	− 0.0134 to 0.036
FTO	0.019210819	− 0.159 to − 0.012
ALKBH5	0.555755013	− 0.077 to 0.042

### Evaluation of the association of erasers and writers

**Fig. 2:**
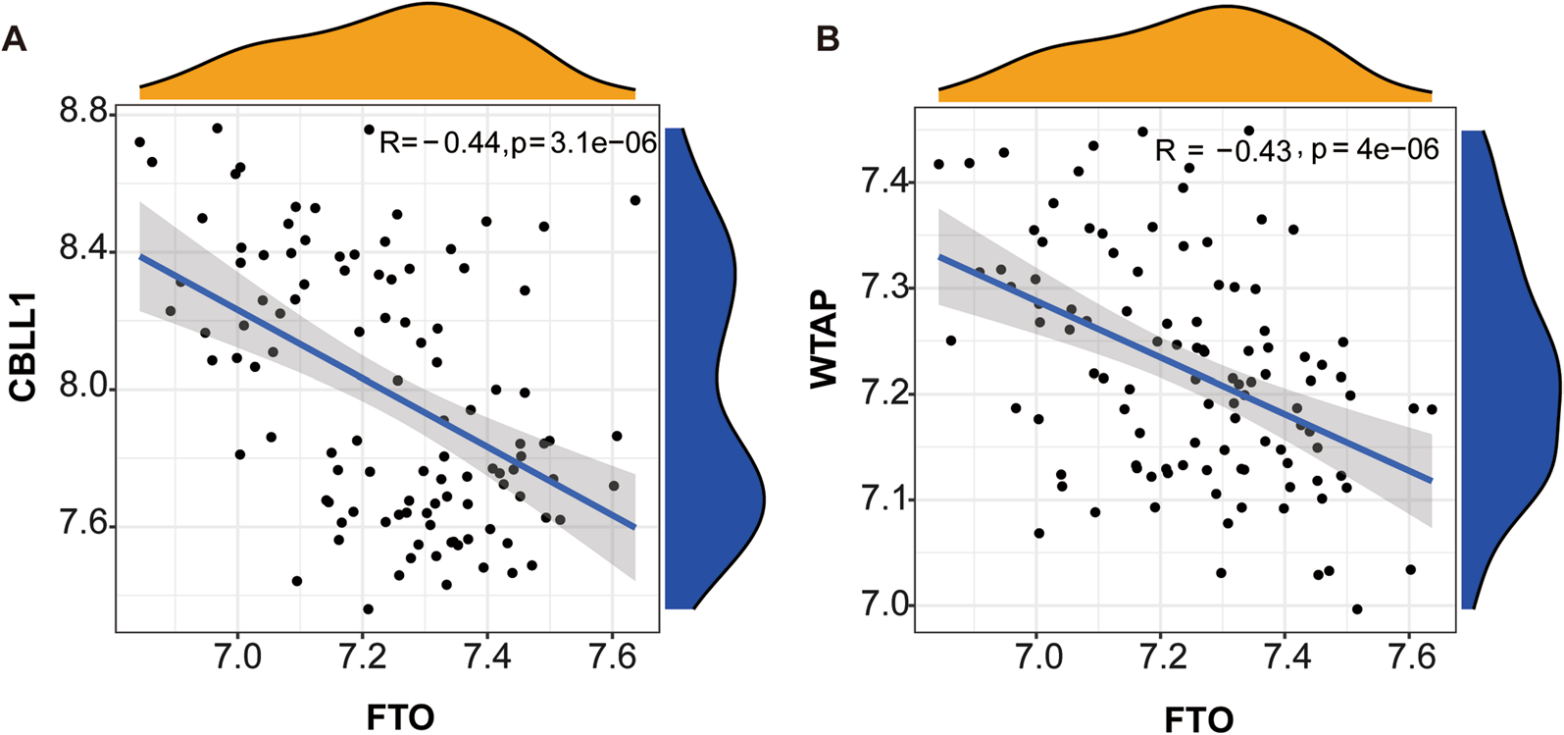
Correlation evaluation of writers and erasers. **A** A negative association was noted between CBLL1 and FTO (R = − 0.44, *p* < 0.001); **B** A negative association was noted between WTAP and FTO (R = − 0.43, *p* < 0.001)

m6A is the most enriched internal modification in the eukaryotes and is engaged in a variety of biological processes [10]. The effect of m6A is primarily mediated through erasers, writers, and readers [5]. The results of linear regression analysis revealed that CBLL1 and FTO were negatively correlated (Fig. 2A), while WTAP and FTO were also negatively correlated (Fig. 2B).

### Model selection

**Fig. 3:**
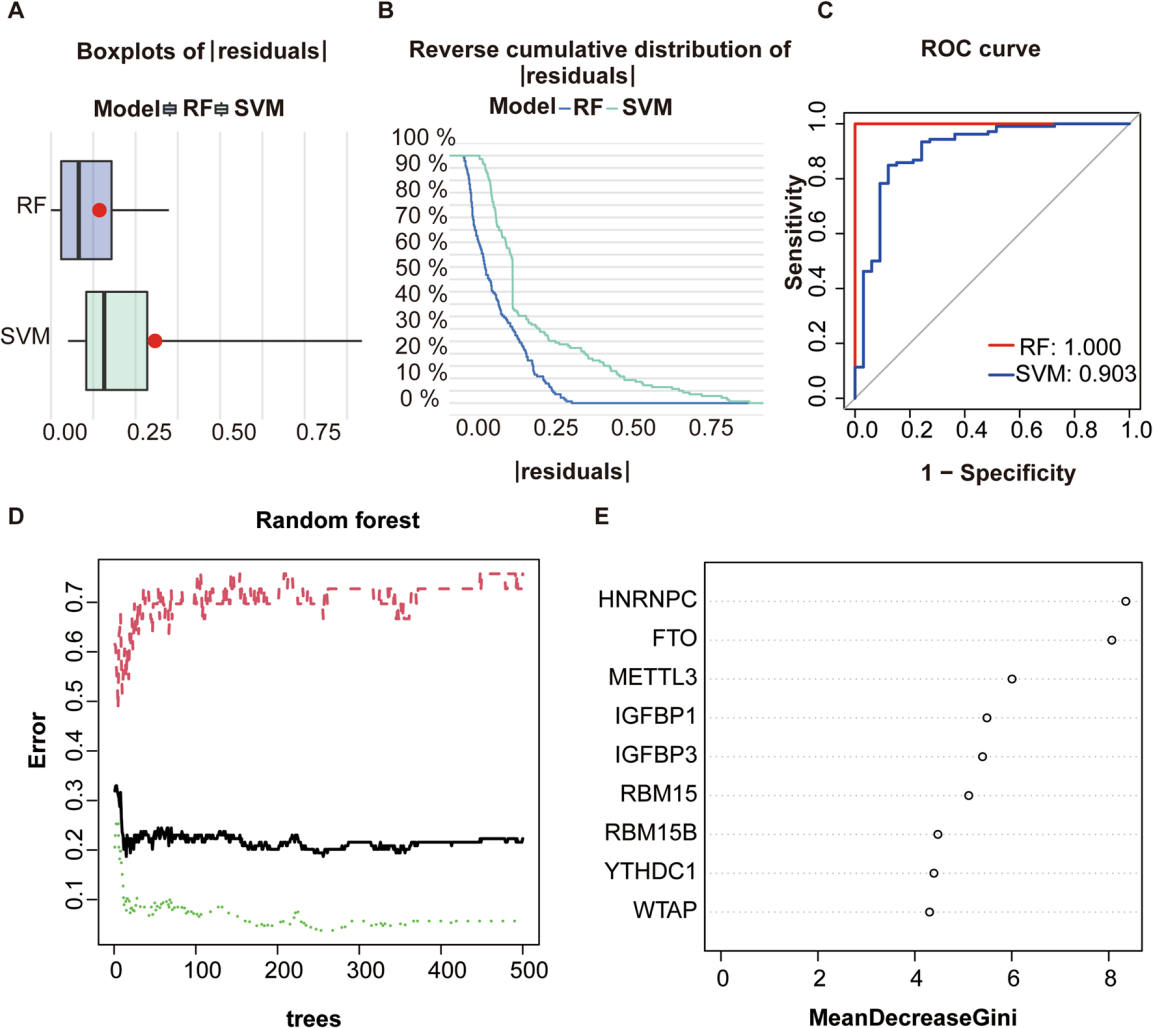
The construction of a RF model. **A** The residuals’ reverse cumulative distribution exhibits the residuals of SVM and RF models, and the red dots represent the residuals’ root mean square; **B** Boxplots of residual reflecting the residuals of SVM and RF models; **C** ROC curves exhibiting the accuracy of SVM and RF models; **D** Outcomes of the random forest plot; **E** Significance score of 9 critical m6A moderators on the basis of a RF model

RF and SVM models were constructed based on the 9 selected m6A regulators. the residuals of the RF model are smaller as compared to that of the SVM model, as illustrated in the “Reverse cumulative distribution of residual” (Fig. 3A) together with “Boxplots of residual” (Fig. 3B). However, the receiver operating characteristics (ROC) curve suggested that the accuracy of the RF model is higher than that of the SVM model (Fig. 3C). Conclusively the RF model is a relatively more ideal model for predicting the risk of osteoarthritis. The results of the RF model are provided in the figure below (Fig. 3D). Regulators were then ranked according to the importance score of the RF model (Fig. 3E). Regulators with an importance score of > 2 were selected for subsequent disease risk assessment.

### Establishment of the nomogram

**Fig. 4:**
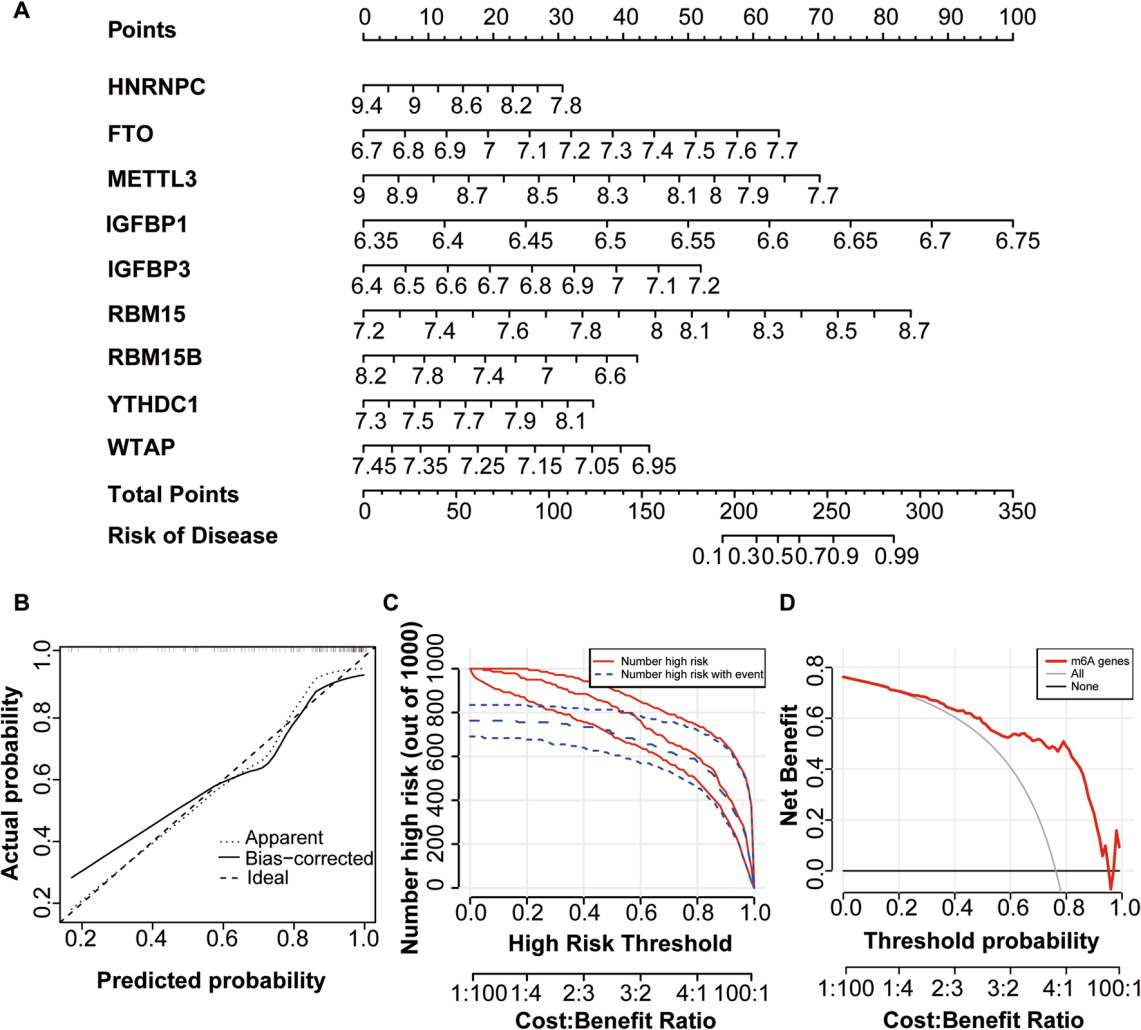
Creation of a nomogram. **A** Nomogram of 9 critical m6A regulators; **B** The calibration curve to assess the nomogram model predicted possibility; **C** The clinical effect for the nomogram model was evaluated with clinical impact curve; **D** The DCA curve reflects the nomogram model accuracy

The nomogram model of the 9 key m6A regulators obtained from the RF model was established to evaluate osteoarthritis disease risk (Fig. 4A). Then the stability of the nomogram model was assessed by calibration curves revealing a high nomogram model accuracy (Fig. 4B). Clinical impact curves show high true-positive rates for high-risk patients, predicted using the nomogram model (Fig. 4C). The decision curve analysis (DCA) curve indicates that the nomogram model has a high accuracy of prediction (Fig. 4D).

### Determination of two subtypes based on 9 critical m6A regulators

**Fig. 5:**
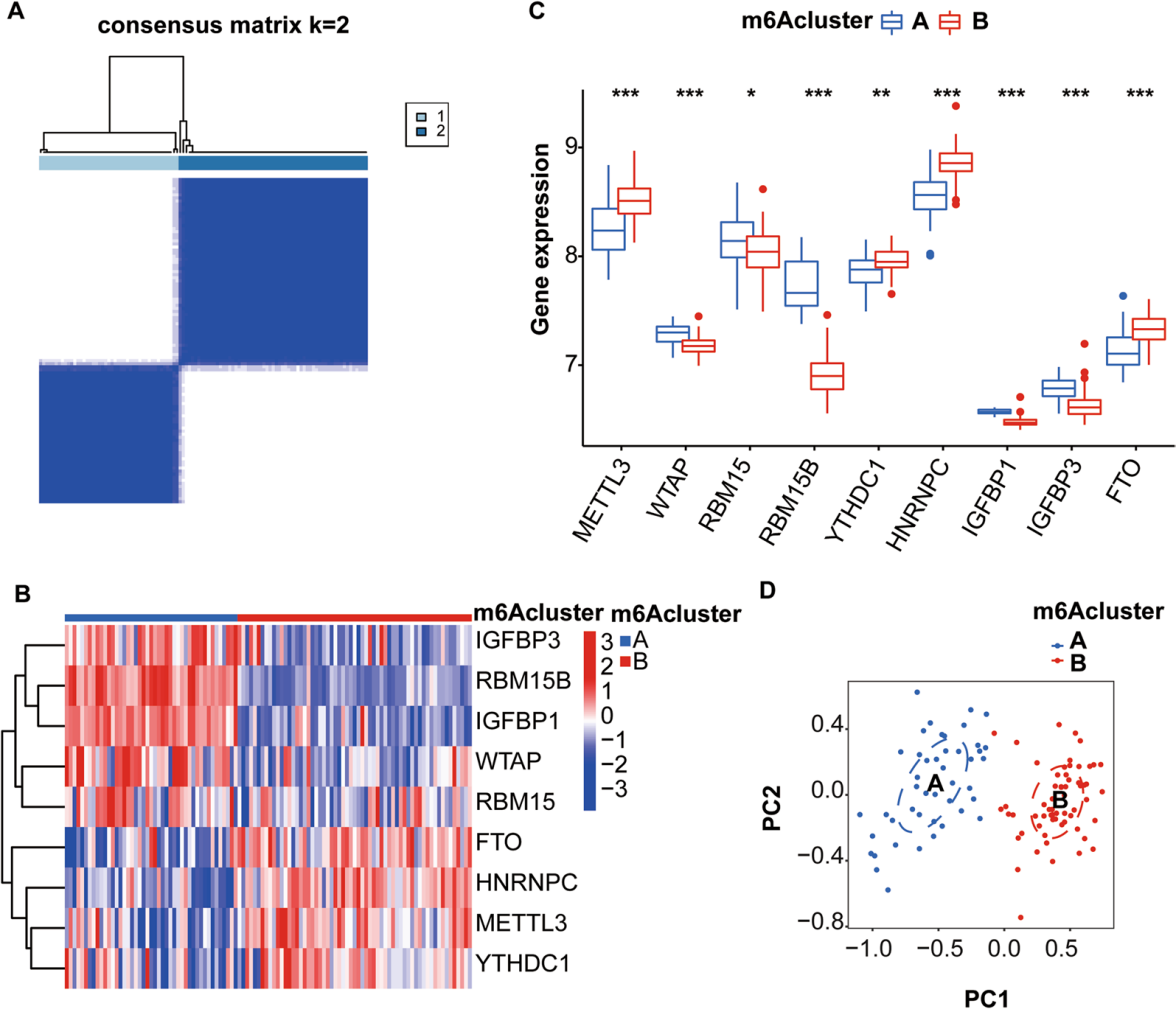
Subgrouping of Osteoarthritis Disease Samples. **A** Consensus clustering of 9 key regulators; **B** Heatmap of differential expression of 9 key regulators in the two subgroups; **C** Histogram of differential expression of 9 key regulators in the two subgroups; **D** PCA of the two subgroup classifications. *p < 0.05, **p < 0.01, and ***p < 0.001

The consensus clustering approach classified osteoarthritis samples into two subtypes- m6Acluster A and m6Acluster B, based on the 9 key m6A regulators (Fig. 5A–D). Cluster A contains 45 samples, and cluster B contains 61 samples. The optimal number of clusters (K) value was determined by consensus cluster analysis, and observed that the consensus is highly stable at a K value of 2 (Fig. 5A). Histograms and heatmaps exhibit the differential expression of the 9 critical m6A regulators in both subgroups (Fig. 5B, C). Cluster A has higher expression levels of IGFBP3, RBM15, WTAP, IGFBP1, and RBM15B in comparison with cluster B, whereas the expression of YTHDC1, METTL3, FTO, and HNRNPC was higher in cluster B than in cluster A. The adjust *p* values and 95% confidence intervals (CI) of the differential expression of the 9 m6A regulators between m6AclusterA and m6AclusterB are shown in Table 2. These 9 key regulators were further confirmed by principal component analysis (PCA) to accurately classify disease samples into two subgroups (Fig. 5D). Therefore, the osteoarthritis disease group was divided into two m6A subgroups based on these 9 key m6A regulators.

**Table 2:** *p* values and confidence intervals for the differential expression of 9 m6A regulators between m6AclusterA and m6AclusterB

Gene	*P* (adj)	95% CI
METTL3	3.06E−07	− 0.351 to − 0.163
WTAP	8.03E−07	0.072 to 0.151
RBM15	0.019814274	0.021 to 0.194
RBM15B	2.12E−18	0.717 to 0.884
YTHDC1	0.001424794	− 0.139 to − 0.039
HNRNPC	3.67E−10	− 0.353 to − 0.204
IGFBP1	6.86E−16	0.085 to 0.109
IGFBP3	3.00E−09	0.119 to 0.204
FTO	1.44E−07	− 0.261 to − 0.132

### Examination of the association between the immune cell infiltration and m6A subtypes

**Fig. 6:**
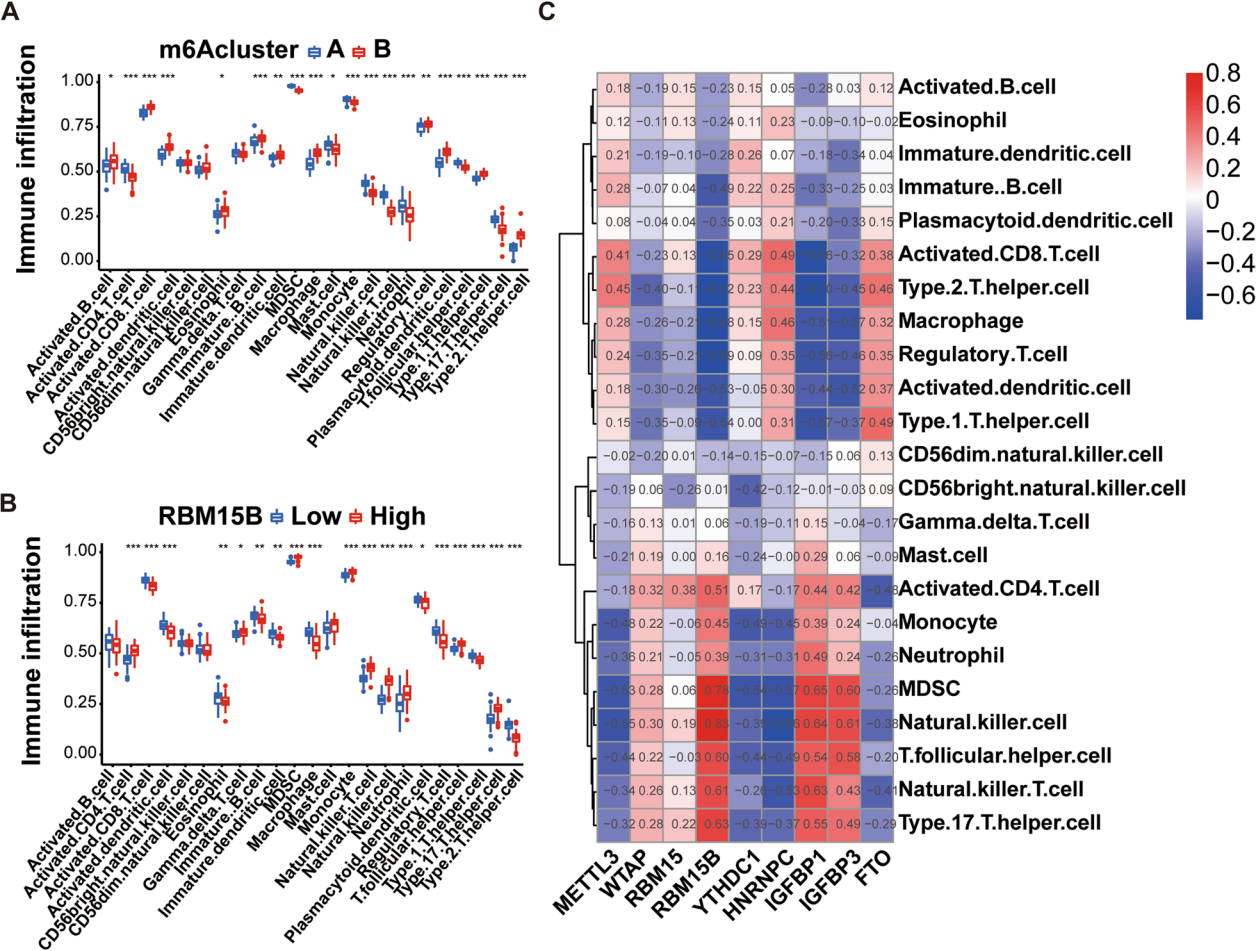
Association analysis of immune infiltration and m6A. **A** Association analysis of immune infiltration and m6A subtypes. **B** Association analysis of immune infiltration and m6A gene. **C** Association analysis of immune infiltration and 9 critical m6A regulators. *p < 0.05, **p < 0.01, and ***p < 0.001

Osteoarthritis manifests as a chronic inflammatory response of the bone and joints. Related studies have revealed that immune response resulting from the infiltration of immune cells participates in the OA damage process [11, 12]. Thus, the differences between the two m6A isoforms were explored in a variety of immune cells, The results showed that the 2 m6A subtypes showed significant differences mainly in the infiltration of immune cells (activated CD8(+)T cell, myeloid-derived suppressor cells, monocyte, immature B cell and plasmacytoid dendritic cell).(Fig. 6A).

In order to study the role and mechanism of immunity in osteoarthritis, we analyzed the relationship between 9 key m6A regulators and immune cell infiltration, among which RBM15B is the m6A regulator most related to immune cell infiltration (Fig. 6C), Suggest that RBM15B is an immune-related regulator in osteoarthritisNext, the case samples of the OA group were then classified into RBM15B low and high expression groups to study the differential expression of a variety of immune cells. The outcomes suggest differences in different immune cells expression between RBM15B low and high expression groups. There were significant differences in the infiltration of immune cells (activated CD8(+) T cell, myeloid-derived suppressor cells, monocyte and activated dendritic cell) among the RBM15B high and low expression groups (Fig. 6B).

### Determination of genotypes based on m6A isoforms

**Fig. 7:**
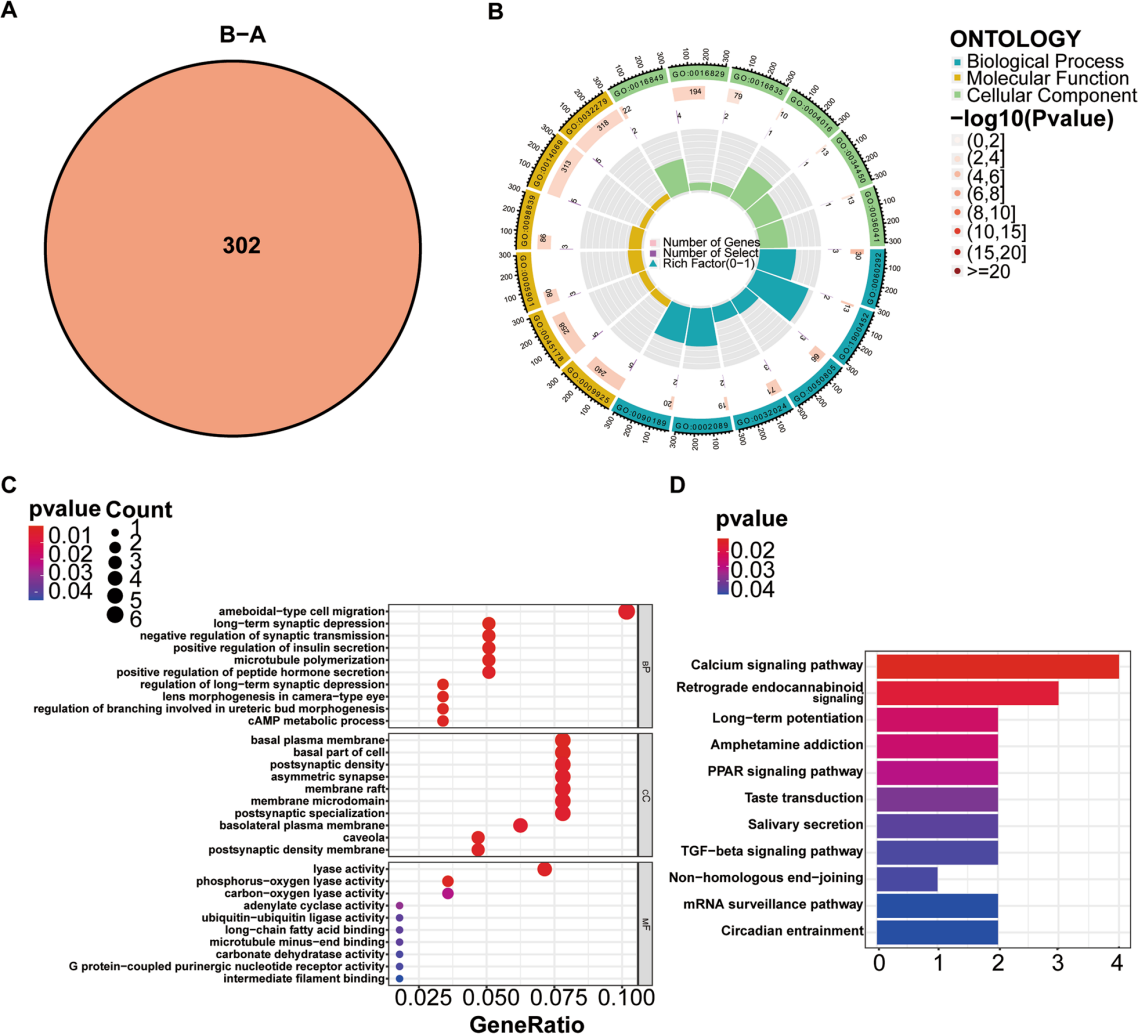
Differential gene expression and GO and KEGG analyses of the m6A isoforms. **A** Venn plot displaying DEGs between both m6A isoforms; **B** Circle plot displaying GO functional enrichment analysis for the DEGs between both m6A isoforms; **C** Bubble plot reflecting GO functional enrichment analysis for the DEGs between both m6A isoforms; **D** Barplot shows the enrichment analysis of DEGs signaling pathways between two m6A subtypes using KEGG [13–15]

**Fig. 8:**
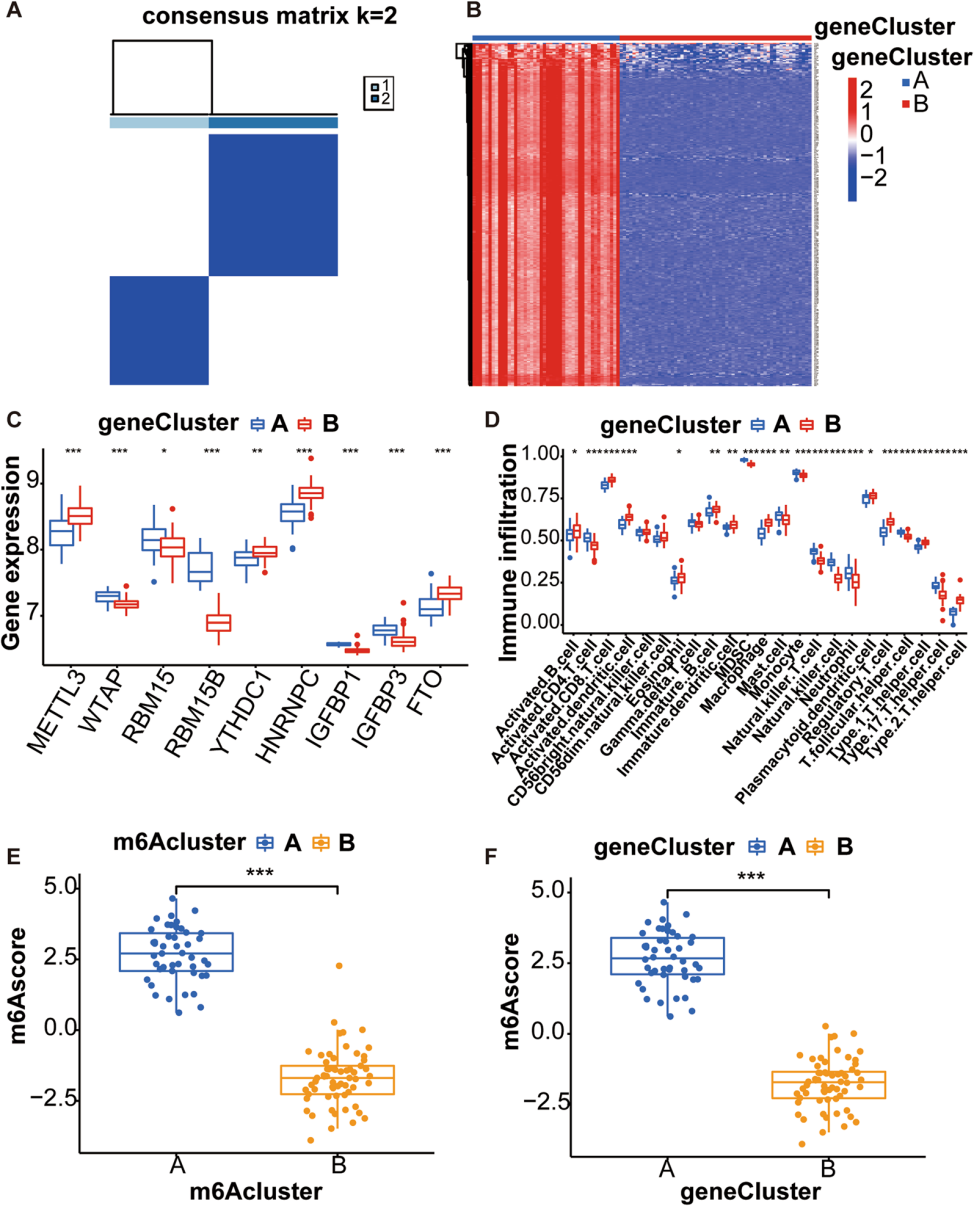
The consensus clustering analysis of DEGs between both m6A isoforms. **A** Consensus clustering analysis separates osteoarthritis patient samples into two genotypes; **B** Heatmap showing differential expression of these 302 genes in two genotypes; **C** Histograms showing differential expression of 9 key m6A regulators between two gene isoforms; **D** Histograms displaying differences in the immune infiltration between both genotypes; **E** Differences of the m6A scores of both m6A subtypes; **F** Differences in m6A scores of the two gene subtypes. *p < 0.05, **p < 0.01, and ***p < 0.001

**Fig. 9:**
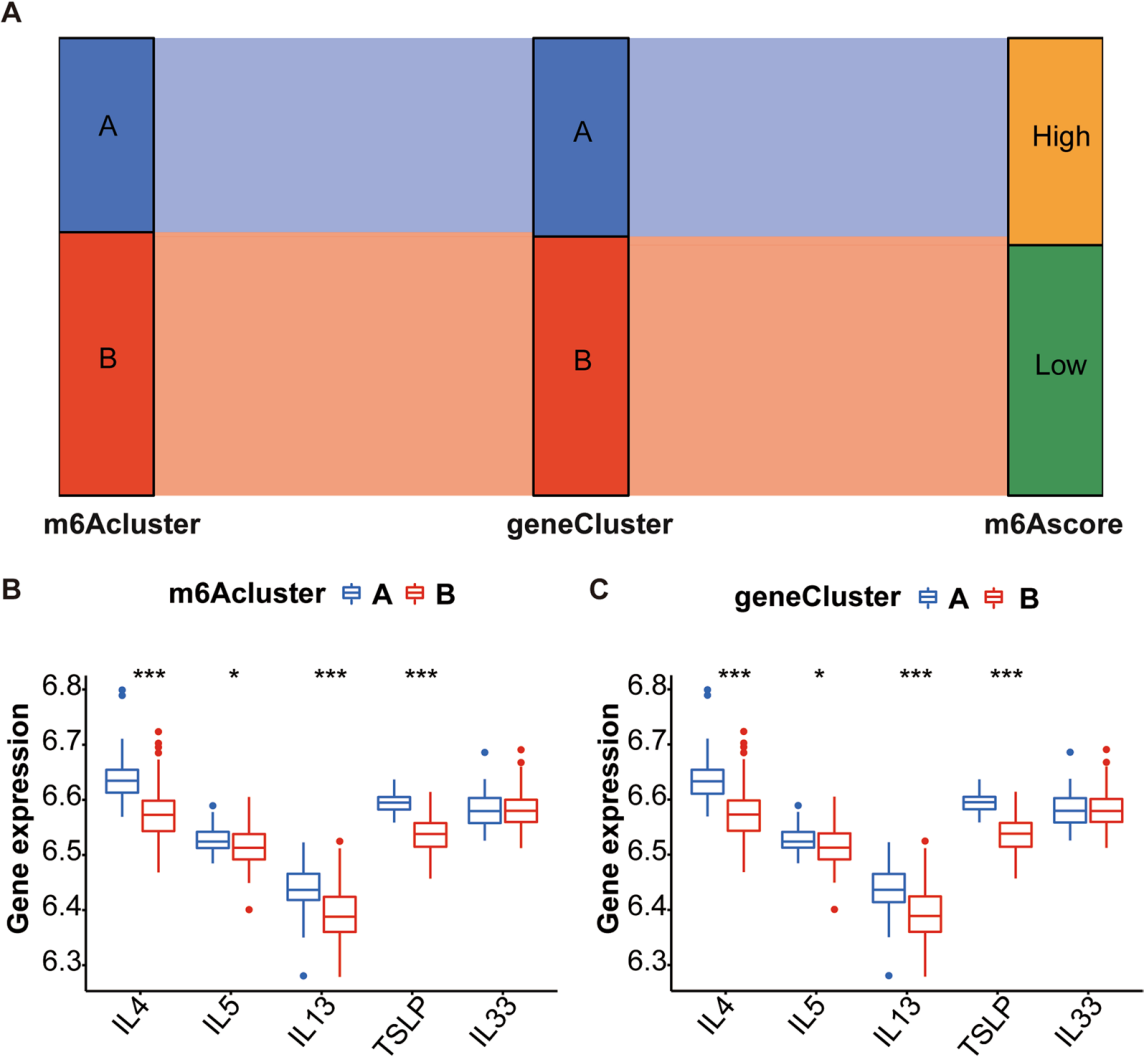
Significance of the m6A pattern in the classification of osteoarthritis. **A** Sankey diagram exhibiting the association between m6A score, genotype and m6A subtype. **B** Differential expression of cytokines between both m6A isoforms. **C** The differences of cytokines between both gene isoforms

Differential gene expression studies between the two subtypes (namely, m6A cluster A and m6A cluster B) resulted in 302 differentially expressed genes (DEGs) (Fig. 7A). Functional enrichment and signaling pathway analysis were implemented according to KEGG and GO databases. Both the GO and KEGG pathways showed significant enrichment of DEGs for ameboidal-type cell migration, basal plasma membrane, basal part of the cell, postsynaptic density, asymmetric synapse, membrane raft, membrane microdomain, postsynaptic specialization, and calcium signaling pathway (Fig. 7B–D). Like the m6A subgroup classification, the consensus clustering approach was used to divide the osteoarthritis disease group into two distinct gene subtypes based on 302 DEGs. Additionally, the two genotypes exhibit similar characteristics as the two m6A subtypes. (Fig. 8A). Differential expression for 302 genes in both genotypes is provided as a heatmap (Fig. 8B). The levels of differential expression for the immune cell infiltration and 9 critical m6A regulators in both genotypes are exhibited as histograms and are similar to the results for the m6A subtype (Fig. 8C, D). The m6A scores of the m6A subtypes and genotypes were analyzed by PCA.From the Sankey diagram in Fig. 9A, we can see that the two typing patterns (m6a typing and gene typing) have a great degree of fitting, so the m6A scores of the two are highly similar. Subtype B (m6AclusterB and geneclusterB) of both typing patterns had higher m6A scores than subtype A (m6AclusterA and geneclusterA) (Fig. 8 E, F).

### Significance of m6A models in diagnosing osteoarthritis

The Sankey diagram revealed that the results of the m6A subtype and genotype classification are similar. However, m6A subtype A and genotype A showed higher m6A scores than m6A subtype B and genotype B (Fig. 9A). We then assessed the differential expression of some cytokines (IL4, IL5, IL13, cytokine thymic stromal lymphopoietin [TSLP] and IL33) between these two m6A subtypes (m6AclusterA and m6AclusterB) (Fig. 9B).

We also performed differential expression of cytokines for two gene subtypes (geneclusterA and geneclusterB) (Fig. 9C). Due to the high degree of fitting of the two typing patterns, the cytokine difference analysis results of the two typing patterns are highly similar. Compared with A subtype (m6AclusterA and geneclusterA) of the two typing patterns, B subtype (m6AclusterB and geneclusterB) had relatively higher expressions of cytokines IL4, IL5, IL13, and TSLP.

## Discussion

Osteoarthritis is the most prevalent chronic rheumatic disease around the world, which seriously influences the health and life quality of people [16]. m6A is the most ubiquitous modification of mRNA in the eukaryotes and exerts an essential effect on growth, development, and disease [17]. m6A modifications are coordinated through m6A-binding proteins (“readers”), demethylases (“erasers”), and m6A methyltransferases (“writers”) [18]. Writers include RBM15, RBM15B, CBLL1, WTAP, METTL3, METTL14, KIAA1429, and ZC3H13; erasers are composed of FTO and ALKBH5; readers include YTHDF3, YTHDF2, YTHDF1, YTHDC2, YTHDC1, FMR1, ELAVL1, HNRNPA2B1, HNRNPC, IGF2BP1, and LRPPRC. Notably, the associated research has revealed that m6A modifications participate in OA pathogenesis and development through modulating pathophysiological processes in cartilage and bone [19]. METTL3 is an m6A methyltransferase that can accelerate aging and osteoarthritis progression by regulating autophagy [7]. It can also modulate the apoptosis and inflammatory response of chondrocytes to affect the process of OA [20]. In conclusion, m6A modification affects the OA occurrence together with its advancement and is of significant importance for the early diagnosis of OA together with its targeted therapy.

In this work, the purpose was primarily to investigate the major clinical importance of m6A modification in diagnosing OA. The differential expression of the m6A regulators in normal and OA human samples was explored through the Gene Expression Omnibus (GEO) database and 9 important differentially expressed m6A regulators (i.e., METTL3, WTAP, RBM15, RBM15B, YTHDC1, HNRNPC, IGFBP1, IGFBP3, and FTO) were identified. However, due to the lack of relevant m6A datasets, we were unable to use independent datasets to validate our typing schema. The risk of OA was predicted by constructing a nomogram model based on these 9 key risk factors. We confirmed the predictive accuracy of the nomogram model by utilizing DCA curves, clinical impact curves, and calibration curves. Then, a consensus clustering algorithm was applied for dividing the OA disease group into 2 distinct m6A subtypes and genotypes, and we found that m6A subtypes and genotypes share this extremely similar feature. OA is a joint disease, and it has the characteristics of joint inflammation and cartilage degeneration [21]. Inflammation is an important characteristic of OA and is correlated with OA symptoms and the development of disease [22]. Therefore, the infiltration degree of the immune cells for both m6A patterns was assessed, and the outcomes indicated that both m6A patterns had significant immune cell infiltration. The inflammatory response is mainly exerted by cytokines. Relevant studies have shown that the helper T-cell (Th cells) response exerts an essential role in OA pathogenesis and OA-related symptoms [23]. Therefore, the Th immune responses (consists of Th1 and Th2 immune responses) are speculated to participate in the disease process of osteoarthritis. The Th1 immune response is composed of interleukin (IL-12, IL-2, interferon-gamma [INF-γ], and tumor necrosis factor-alpha [TNF-α]), while the Th2 immune response is exerted by IL-13, IL-10, IL-6, IL-5, IL- 4, and cytokine thymic stromal lymphopoietin [TSLP] [24]. We scored the two m6A patterns with the help of the PCA algorithm and assessed the degree of Th immune response in the two subtypes. The results showed that the m6A pattern A had a higher m6A score than the m6A pattern B and that the m6A pattern A had a relatively higher expression of the Th2 immune response levels (i.e., IL-4, IL-5, IL-13, TSLP). Therefore, we inferred that the m6A pattern A has a higher risk of developing osteoarthritis, which exerts its pathogenic role through the Th2 immune response. The typing outcomes of this research can be considered the basis for future studies on pathogenic mechanisms associated with specific m6A. In addition, this study findings provide a new OA diagnostic biomarker and therapeutic molecular target for clinical studies.

## Conclusion

In conclusion, we screened nine important m6A regulators and constructed a disease risk prediction nomogram model based on the random forest algorithm. This model has been verified to accurately predict the risk of osteoarthritis. At the same time, we found that m6A pattern A may be associated with a higher risk of osteoarthritis.

## Methods

### Data acquisition

From the GEO database, the dataset of GSE48556 could be acquired, which included 33 normal human samples and 106 osteoarthritis patient samples. The clinicopathological data of the patients was presented in the table (Additional file 1: Table S1). The detailed clinicopathological data of the patients included in the GEO data can be found in the literature [25].A total of 21 m6A regulators contained 11 readers (namely, YTHDF1, YTHDF2, YTHDF3, YTHDC1, YTHDC2, FMR1, ELAVL1, HNRNPA2B1, HNRNPC, IGF2BP1, and LRPPRC), 2 erasers (i.e., FTO and ALKBH5), and 8 writers (namely, WTAP, CBLL1, KIAA1429, METTL14, METTL3, RBM15B, RBM15, and ZC3H13) [26].

### Random forest (RF) and support vector machine (SVM) models

In the present study, SVM together with RF models were used to evaluate the risk of developing osteoarthritis. RF is a commonly used ensemble learning algorithm, and its base classifier is a decision tree. We completed the RF algorithm through the “randomForest” package in R software. The ntree and mtry parameters of the RF model were 500 and 3, respectively. We also screened 9 key regulators from 21 m6A regulators based on the gene importance score. The filter criterion was the importance score value > 2. SVM algorithm is a type of machine-learning approach based on the statistical learning theory. It enhances the generalization ability for learning machine by seeking to minimize the confidence range, empirical risk, and structural risk in order to realize the goal of obtaining favorable statistical laws even with a small statistical sample size. Through the curve analysis of receiver operating characteristic (ROC), "boxplots of residuals", and "reverse cumulative distribution of residuals", the accuracy for both the models were assessed.

### Nomogram model

We used the “rms” and “rmda” packages in R to construct the nomogram model for application in predicting the risk for OA. Decision curve analysis (DCA) and calibration curves were used to assess the nomogram model predictive accuracy. In addition, whether the nomogram model predictions could benefit patients was assessed through clinical impact curves.

### Identification of 2 m6A isoforms and m6A gene isoforms

The consensus clustering analysis was implemented on the sample data from the database of GEO according to 21 m6A regulators, and the sample data was classified as m6A models. Differentially expressed genes (DEGs) associated with m6A were screened through differential expression among the m6A heterodimers. Then, the DEGs related to m6A subtypes were classified into different m6A subtypes through consensus clustering analysis. DEGs associated with m6A were realized via the “limma” package in R. The consensus clustering algorithm for subtype classification was implemented by the “ConsensusClusterPlus” package of R software.

### The m6A scoring of the sample

To quantitatively analyze the m6A modification pattern of osteoarthritis, we employed the m6A score to evaluate the gene signature of the m6A pattern of osteoarthritis. Accordingly, we calculated the m6A score through principal component analysis, a widely applied dimensionality reduction algorithm for data. Initially, PCA was utilized to determine the m6A pattern and next the m6A score was counted in accordance with the below mentioned formula: m6A score = 6 (PC1i + PC2i), wherein PC1 and PC2 denoted the principal component 1 and 2, respectively, and i indicate the DEGs associated with m6A [27].

### Kyoto Encyclopedia of Genes and Genomes (KEGG) together with Gene Ontology (GO) pathway for the analysis of DEGs between various m6A patterns

To explore the DEGs functional enrichment between various m6A patterns, KEGG along with the GO pathway analyses were implemented on these genes. The GO enrichment analysis was composed of cellular component (CC), molecular function (MF), and biological process (BP). The pathway analysis of KEGG displays potential signaling pathways [28].

### Assessment of the immune-infiltrating microenvironment

For the immune cells, the extent of infiltration in the samples of OA was evaluated with ssGSEA. ssGSEA was applied for ranking the levels of gene expression in the samples and acquiring their grades. Subsequently, these genes were retrieved from the dataset and the expression levels of these genes were summed. Finally, we evaluated the infiltration degree of the immune cells in each sample [29].

### Statistical analysis

The association between writers and erasers was investigated with linear regression analysis. Kruskal–Wallis test and Wilcoxon test were utilized for the comparison of differences among multiple groups and between 2 groups, respectively. On the basis of two-tailed test, all parametric analyses were conducted, and p < 0.05 was considered to indicate a statistical significance. The R version 4.1.3 was employed for conducting all statistical analyses.

## Supplementary Information

**Additional file 1: Table S1.** The information of the samples

## Data Availability

The data used in this study are available on the GEO database(https://www.ncbi.nlm.nih.gov/geo/) with accession number GSE48556.
